# Optimizing PO_2_ during peripheral veno-arterial ECMO: a narrative review

**DOI:** 10.1186/s13054-022-04102-0

**Published:** 2022-07-26

**Authors:** Hadrien Winiszewski, Pierre-Grégoire Guinot, Matthieu Schmidt, Guillaume Besch, Gael Piton, Andrea Perrotti, Roberto Lorusso, Antoine Kimmoun, Gilles Capellier

**Affiliations:** 1 Service de Réanimation Médicale, centre hospitalier universitaire de Besançon, Besançon, France; 2 Service d’Anesthésie-Réanimation Chirurgicale, centre hospitalier universitaire de Dijon, Dijon, France; 3 Service de Médecine Intensive Réanimation, Institut de Cardiologie, APHP Sorbonne Université Hôpital Pitié-Salpêtrière, Paris, France; 4 Service d’Anesthésie-Réanimation Chirurgicale, centre hospitalier universitaire de Besançon, Besançon, France; 5 Service de Chirurgie Cardiaque, centre hospitalier universitaire de Besançon, Besançon, France; 6 Cardio-Thoracic Surgery Department, Maastricht University Medical Centre (MUMC), Cardiovascular Research Institute Maastricht (CARIM), Maastricht, The Netherlands; 7 Service de Médecine Intensive Réanimation, centre hospitalier universitaire de Nancy Brabois, Vandœuvre-lès-Nancy, France; 8 Department of Epidemiology and Preventive Medicine, School of Public Health and Preventive Medicine, Faculty of Medicine, Nursing and Health Sciences, Clayton, Australia; 9 Research Unit EA 3920 and SFR FED 4234, University of Franche Comté, Besancon, France

**Keywords:** Veno-arterial ECMO, Oxygen, Hyperoxemia, Dual circulation, Mixing zone

## Abstract

During refractory cardiogenic shock and cardiac arrest, veno-arterial extracorporeal membrane oxygenation (VA-ECMO) is used to restore a circulatory output. However, it also impacts significantly arterial oxygenation. Recent guidelines of the *Extracorporeal Life Support Organization* (ELSO) recommend targeting postoxygenator partial pressure of oxygen (P_POST_O_2_) around 150 mmHg. In this narrative review, we intend to summarize the rationale and evidence for this P_POST_O_2_ target recommendation. Because this is the most used configuration, we focus on peripheral VA-ECMO. To date, clinicians do not know how to set the sweep gas oxygen fraction (F_S_O_2_). Because of the oxygenator’s performance, arterial hyperoxemia is common during VA-ECMO support. Interpretation of oxygenation is complex in this setting because of the dual circulation phenomenon, depending on both the native cardiac output and the VA-ECMO blood flow. Such dual circulation results in dual oxygenation, with heterogeneous oxygen partial pressure (PO_2_) along the aorta, and heterogeneous oxygenation between organs, depending on the mixing zone location. Data regarding oxygenation during VA-ECMO are scarce, but several observational studies have reported an association between hyperoxemia and mortality, especially after refractory cardiac arrest. While hyperoxemia should be avoided, there are also more and more studies in non-ECMO patients suggesting the harm of a too restrictive oxygenation strategy. Finally, setting F_S_O_2_ to target strict normoxemia is challenging because continuous monitoring of postoxygenator oxygen saturation is not widely available. The threshold of P_POST_O_2_ around 150 mmHg is supported by limited evidence but aims at respecting a safe margin, avoiding both hypoxemia and severe hyperoxemia.

## Background

During cardiogenic shock or cardiac arrest refractory to medical treatment, peripheral veno-arterial extracorporeal membrane oxygenation (VA-ECMO) is used to restore adequate oxygen delivery, mainly by increasing systemic blood flow. However, the oxygenator integrated to the VA-ECMO circuit also impacts arterial oxygen saturation of hemoglobin (S_a_O_2_) and arterial oxygen partial pressure (P_a_O_2_). If the ECMO blood flow management can be guided by lactate and mixed venous oxygen saturation in the pulmonary artery (S_v_O_2_) [1], data to guide the sweep gas oxygen fraction (F_s_O_2_) management are scarce.

In the recent *Extracorporeal Life Support Organization (ELSO) Interim Guidelines for Venoarterial Extracorporeal Membrane Oxygenation in Adult Cardiac Patients*, the experts stated that “excessive hypo- and hyperoxemia should be avoided” and that “gas blender should be adjusted to target slight hyperoxemia after the oxygenator (150 mmHg)” [1]. However, no ideal range for oxygenation is provided, and these recommendations open the F_s_O_2_ setting to large variations in the VA-ECMO practices. While some data support the harm of severe hyperoxemia [2], recent randomized studies on non-ECMO patients with acute respiratory distress syndrome and sepsis raise concern about the potential risk of a restrictive oxygenation strategy [3–5].

In this review, we aim at summarizing the rationale, evidence, and limits of the recent postoxygenator PO_2_ (P_POST_O_2_) target recommendation. Because it is the most used configuration, we focus on peripheral VA-ECMO. As so, pathophysiological concepts developed herein are not strictly transposable to central VA-ECMO. Also, while CO_2_ management during VA-ECMO seems to be another key issue, especially with the risk of hypocapnia [6, 7], it deserves a special focus and is not developed herein.

## PO_2_ during peripheral VA-ECMO support: what are we talking about?

### Definitions

During VA-ECMO support, several factors impact patient oxygen delivery: hemoglobin level, native lung function, oxygenator, native cardiac output, and ECMO blood flow.

**Fig. 1:**
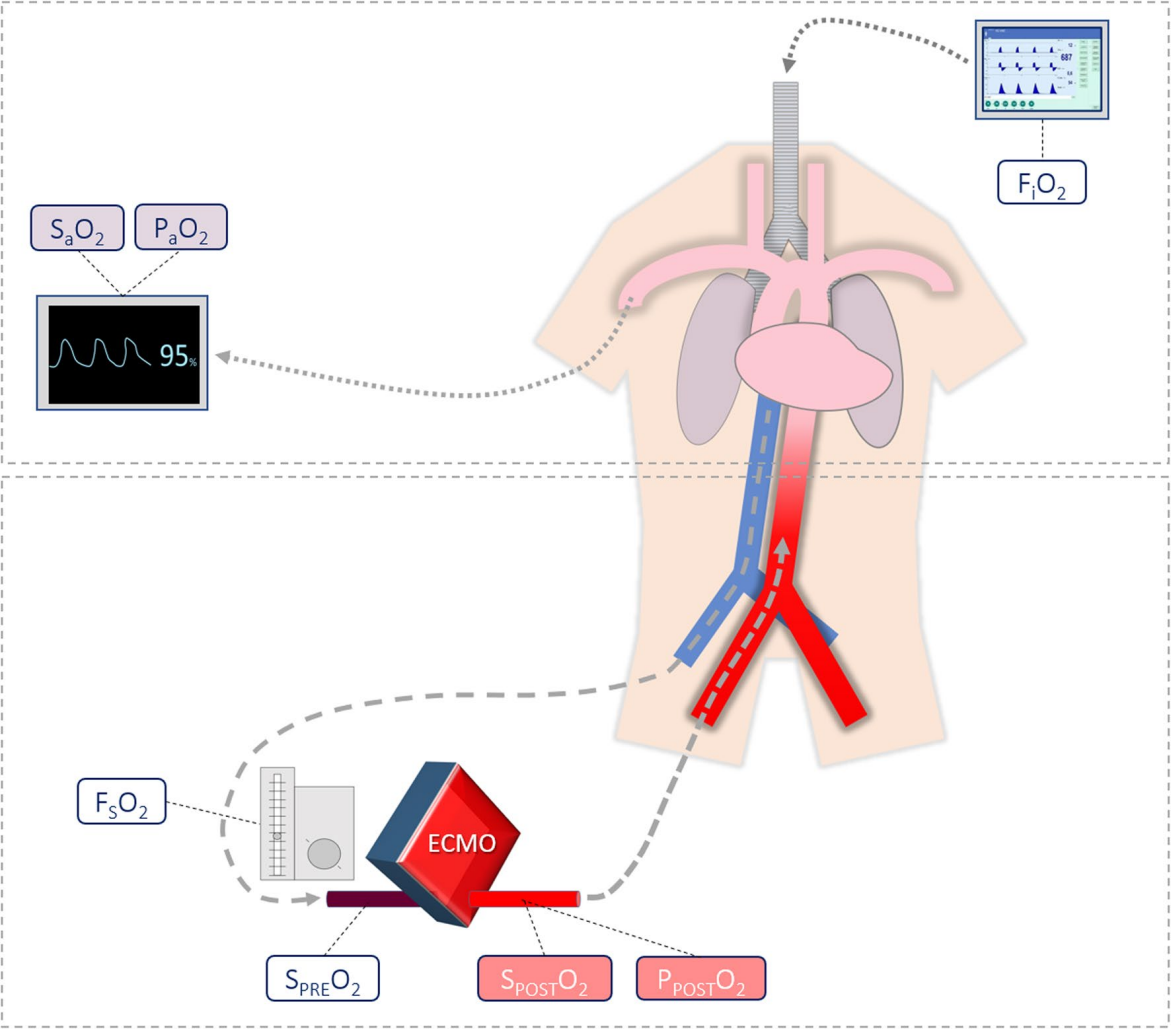
Oxygenation parameters during peripheral VA-ECMO support. S_a_O_2_: arterial oxygen saturation of hemoglobin; P_a_O_2_: arterial partial pressure of oxygen; F_I_O_2_: inspired oxygen fraction; F_S_O_2_: sweep gas oxygen fraction; S_PRE_O_2_: preoxygenator oxygen saturation of hemoglobin; S_POST_O_2_: postoxygenator oxygen saturation of hemoglobin; P_POST_O_2_: postoxygenator oxygen partial pressure

In this review, we focus on extracorporeal oxygenation which corresponds to both oxygen partial pressure, and oxygen saturation of hemoglobin just after the oxygenator (*i.e.,* P_POST_O_2_ and S_POST_O_2_, respectively). Measurement of these parameters needs to sample blood gas on the arterial side of the circuit, after the oxygenator. P_POST_O_2_ and S_POST_O_2_ depend on oxygenator gas transfer, which determinants are oxygen saturation of hemoglobin on venous blood before oxygenator (S_PRE_O_2_), hemoglobin concentration, ECMO blood flow, sweep gas oxygen fraction (F_S_O_2_), sweep gas flow, and the oxygenator function. Managing F_S_O_2_ needs to incorporate a gas blender on the sweep gas flow circuit (Fig. 1), allowing titration of air and oxygen mixture. Of note, by itself the sweep gas flow little impacts P_POST_O_2_, whereas it is a major determinant of P_a_CO_2_ by influencing the amount of extracorporeal CO_2_ removal.

Extracorporeal oxygenation must be distinguished from the brain and coronary oxygenation, which surrogates are classically the right radial P_a_O_2_ and S_a_O_2_. These parameters are dependent on several factors: S_v_O_2_, hemoglobin concentration, native lung function, inspired oxygen fraction on ventilator (F_I_O_2_), positive end expiratory pressure, extracorporeal oxygenation, and ratio between native cardiovascular/lung function and the VA-ECMO support.

An overview of the main oxygenation-related parameters during peripheral VA-ECMO support is provided in Fig. 1.

### Dual oxygenation and mixing zone during femoro-femoral VA-ECMO

During femoro-femoral VA-ECMO, while hemodynamic is easily monitored (ECMO blood flow, arterial pressure, etc.), adequate tissue oxygenation monitoring is more challenging. In contrast to the literature on veno-venous ECMO, strong data on oxygenation determinants during VA-ECMO support are lacking [8].

The challenge of oxygenation during femoro-femoral VA-ECMO support is related to the dual oxygenation phenomenon [9], also known as differential hypoxemia, “North–South syndrome,” or “Harlequin syndrome.”

The dual oxygenation phenomenon is linked to the dual circulation phenomenon. During femoro-femoral VA-ECMO configuration, two distinct circulations occur: the native circulation, corresponding to the residual cardiac blood flow, and the extracorporeal circulation. Schematically, in the presence of significant residual cardiac output, the first aortic branches (*i.e.,* the brachio-cephalic trunk and the common left carotid artery) and the upper part of the body (heart and brain) are perfused by the heart and oxygenated by the native lung. The lower part of the body (*i.e.,* gut, liver, kidney, etc.) is perfused by the ECMO flow and oxygenated by the oxygenator. The zone where the two circulations meet is called the mixing zone. Of note, the dual oxygenation phenomenon varies over time. Indeed, the location of the mixing zone, and so the oxygenation level along the aorta, varies according to the degree of VA-ECMO support and the degree of heart impairment [10, 11]. In other words, the higher the ECMO blood flow, the proximal the mixing zone in the aorta. Besides, the lower the native heart ejection, the proximal the mixing zone in the aorta.

**Fig. 2:**
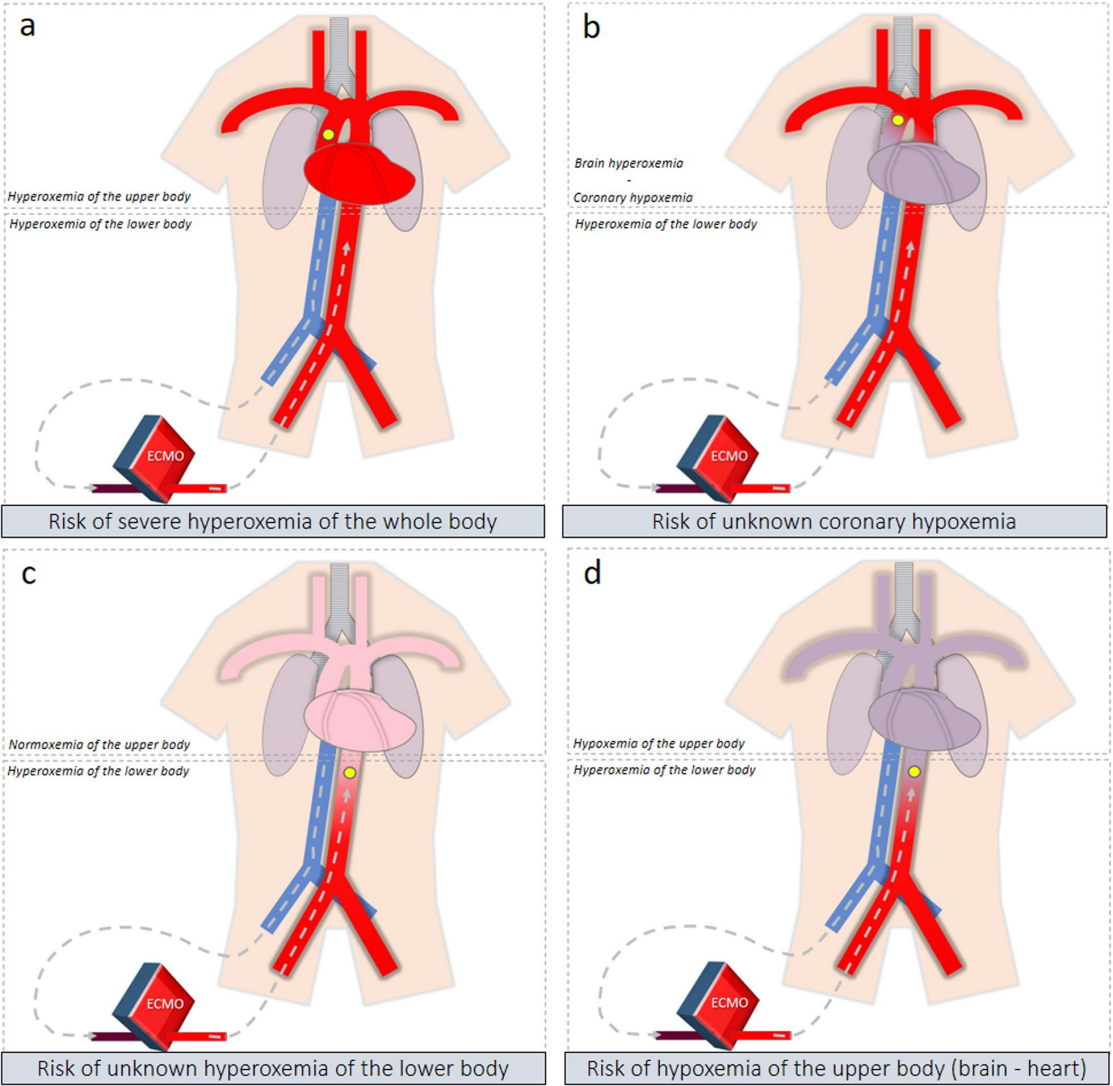
Clinical pictures illustrating the challenge of oxygenation during peripheral VA-ECMO. The yellow bullet corresponds to the mixing zone location; **a** When heart function is severely impaired, the mixing zone is in the proximal aorta and the risk is severe hyperoxemia of the whole body; **b** If there is minimal residual stroke volume and severe lung impairment, the mixing zone is above the coronary arteries but below the brachio-cephalic trunk. Then, the risk is unknown coronary hypoxemia; **c** When the heart recovers, the mixing zone moves down in the descending aorta. The risk is unknown hyperoxemia because continuous monitoring of P_POST_O_2_ is not widely available; **d** When the mixing zone is in the descending aorta, if severe lung impairment is associated, the risk is fulminant differential hypoxemia (Harlequin syndrome) with severe coronary and brain hypoxemia

During the early phase of resuscitation, VA-ECMO is responsible for near-total hemodynamic support because of cardiogenic shock (high ratio between VA-ECMO blood flow and native cardiac output). In such a situation, the mixing zone is proximal in the aortic arch, and VA-ECMO might be responsible for the oxygenation of the near whole body (Fig. 2a). It should be noted, however, that, at this phase, there is specific concern about possible misdiagnosed coronary hypoxemia (Fig. 2b). Indeed, discrepancies between right radial P_a_O_2_ and proximal aorta P_a_O_2_ have been described in the setting of peripheral VA-ECMO [12]. Unknown coronary hypoxemia could be particularly deleterious at the myocardial recovery phase.

Later, as the heart recovers, the native cardiac output increases and the VA-ECMO flow can be decreased. The mixing zone moves down in the descending aorta. At this phase, organ oxygenation assessment is more challenging. When oxygenation is monitored at the right radial artery, P_a_O_2_ and S_a_O_2_ only reflect oxygenation of the upper part of the body. In this situation, physician cannot exclude severe hyperoxemia of the lower part of the body (Fig. 2c). Because P_POST_O_2_ is not continuously monitored, unknown hypoxemia of the lower part of the body is also theoretically possible in case of oxygenator dysfunction.

Finally, it should be noted that left ventricle unloading with an Impella® also contributes to move down the mixing zone in the aorta.

### Relation between extracorporeal oxygenation and systemic oxygenation during femoro-femoral VA-ECMO

While extracorporeal oxygenation firstly impacts oxygenation of the lower part of the body, it might also affect the brain and coronary oxygenation, which clinical surrogates are right radial artery P_a_O_2_ and S_a_O_2_.

As previously exposed, during the early phase of VA-ECMO support, the mixing zone is proximal in the aortic arch, and systemic oxygenation is mainly ensured by the oxygenator [10]. With an F_S_O_2_ commonly set at 100% [13–15] for current oxygenator’s characteristics, P_POST_O_2_ can rise to 500 mmHg at the membrane lung outlet [16]; thus, hyperoxemia is frequently observed on arterial blood gases sampled at the right radial artery [13, 15, 17–20]. In such setting, VA-ECMO can be responsible for brain and coronary hyperoxemia (Fig. 2a).

When the heart recovers, VA-ECMO might also improve brain and coronary oxygenation, through an increase in the S_v_O_2_. It might be clinically relevant in the case of a fulminant differential hypoxemia phenomenon (Fig. 2d). Such a situation appears when the recovering heart ejects severely deoxygenated blood coming from the impaired lungs (for example due to pneumonia). As the blood P_a_O_2_ ejected from the left ventricle is very low, the venous oxygen saturation of the upper part of the body measured in the superior vena cava (S_SVC_O_2_) is very low. If the venous cannula drains blood from the inferior vena cava (IVC) and not from the superior vena cava (SVC), the low S_SVC_O_2_ will result in low S_v_O_2_ and finally lead to a lower S_a_O_2_ in the aortic root. On the opposite, if the venous cannula tip is moved toward the SVC, deoxygenated blood from the SVC will be drained preferentially by VA-ECMO and oxygenated by the membrane lung. Then, S_v_O_2_ will be determined mainly by oxygen saturation of the IVC blood (S_IVC_O_2_). As returning from intra-abdominal organs oxygenated by VA-ECMO, it will be moderately deoxygenated, allowing an increased S_v_O_2_ and finally increased S_a_O_2_ [9, 21–23]. In an experimental study on 15 patients supported by VA-ECMO but without Harlequin syndrome, shifting the drainage cannula tip from IVC to SVC increased right radial P_a_O_2_ from 127 to 153 mmHg [23]. Then, clinical impact of this moderate oxygenation improvement in case of fulminant differential hypoxemia remains to be determined.

### Specificities of femoro-subclavian VA-ECMO

When subclavian (or axillary) artery is preferred for the arterial access, there is no more concern about differential hypoxemia. Indeed, in such a situation, blood oxygenated by the membrane easily reaches the arch vessels, preventing an upper body hypoxemia. Conversion from femoral to subclavian approach is even a therapeutic option in case of severe differential hypoxemia [9].

While of potential interest, it should be noted on the other hand that this configuration may expose brain to hyperoxemia, especially if the right subclavian artery is cannulated, because of its connection with the right common carotid artery. Finally, as with femoro-femoral configuration, there is still a risk of misdiagnosed coronary hypoxemia if the mixing zone is below the brachio-cephalic trunk.

## What is recommended for extracorporeal oxygenation management?

Until 2021, ELSO guidelines did not provide any recommendation about F_S_O_2_, P_POST_O_2_ and S_POST_O_2_ [16]. The recent *ELSO Interim Guidelines for Venoarterial Extracorporeal Membrane Oxygenation in Adult Cardiac Patients* addressed these points. The experts suggest that “excessive hypo- and hyperoxemia should be avoided.” Despite scarce evidence, they further suggest that “gas blender should be managed to target slight hyperoxemia after the oxygenator (150 mmHg)” [1]. These recommendations do not specify lower and upper limits for P_POST_O_2_. The experts recommended also monitoring right radial P_a_O_2_ to detect differential hypoxemia, but without mentioning P_a_O_2_ targets.

Regarding the use of VA-ECMO in resuscitation (ECPR), the recent guidelines do not provide clear recommendation on extracorporeal oxygenation. In the *ELSO Interim Guidelines for Extracorporeal Cardiopulmonary Resuscitation in adults*, the experts state that “Avoidance of hyperoxia can be achieved through the careful blending of ECMO fresh gas flow with an air and oxygen mix.” They recommend “targeting a patient arterial oxygen saturation of 92–97%” without precision on the monitoring site [24]. Finally, guidelines on postcardiotomy ECMO do not provide any recommendation on extracorporeal oxygenation [25].

## What do we know about daily practice?

Reliable data on extracorporeal oxygenation during VA-ECMO support should include F_S_O_2_, P_POST_O_2_ and right radial P_a_O_2_. Although some data regarding to F_S_O_2_ and P_a_O_2_ are available, no study has specifically focused on P_POST_O_2_.

### F_S_O_2_ settings

In a retrospective study on 52 VA-ECMO patients, Justus et al. described the evolution of F_S_O_2_ during the entire ECMO runs. At baseline, median F_S_O_2_ ranged from 72% (interquartile range (IQR) 62–82) to 78% (IQR 70–87). Mean F_S_O_2_ was around 80% between day 1 and day 10 and decreased around 60% between day 10 and day 20 of ECMO support [20]. In a retrospective cohort of 240 VA-ECMO patients evaluating the effect of levosimendan, Distelmaier et al. reported a median F_S_O_2_ at day 1 of 65% (IQR 60–90) in the levosimendan group and 70% (IQR 60–100) in the control group [26]. In another retrospective study on awake VA-ECMO (*n* = 57), Ellouze et al. reported a mean F_S_O_2_ (± standard deviation) of 66% (± 14) in the extubated group at the day of extubation and of 71% (± 17) in the non-extubated group on the third day of ECMO support [27]. Such description of F_S_O_2_ management is rare, and available information regarding F_S_O_2_ mainly comes from institutional protocols described in observational studies. Ross et al. reported that they always maintain F_S_O_2_ at 100% [13]. In the context of ECPR, Lamhaut et al. set the F_S_O_2_ at 50% immediately after ECMO start [28], while Chang et al. set F_S_O_2_ at 60% [17] and Halter and Stoll at 100% [14, 15]. Taking together these studies, F_S_O_2_ is usually set between 50 and 100% during the early phase of VA-ECMO support.

### P_POST_O_2_ and P_a_O_2_

No study specifically provide data on P_POST_O_2_. However, studies describing general oxygenation in VA-ECMO patients could provide some information. Using a threshold of PaO_2_ ≥ 300 mmHg, the reported prevalence of severe hyperoxemia in the first 24 h ranged from 12 to 89% [13–15, 17–19, 29] (Table 1). In the study of Justus et al., the mean right radial P_a_O_2_ was higher than 250 mmHg at day 1 and decreased between day 3 and day 10, ranging from 100 to 150 mmHg [20]. In a retrospective study of 79 ECPR patients, the mean right radial P_a_O_2_ over the 8 first days was 211 ± 58 mmHg [15]. Based on a study on ECPR, the median value of mean P_a_O_2_ in the non-cannulated femoral artery during the first day was 328 mmHg (IQR 228–524) [19].

**Table 1:** Summary of studies reporting outcome associated with hyperoxemia during VA-ECMO support in adults

Studies	Indication for ECMO		Metrics of hyperoxemia	Site of arterial blood gas sampling	Prevalence of severe hyperoxemia (P_a_O_2_ > 300 mmHg)	Impact of hyperoxemia
	CS	ECPR				
Munshi et al. [29]	775	412	P_a_O_2_ 24 h after ECMO initiation	Not available	CS: 15% ECPR: 22%	P_a_O_2_ between 101 and 300 mmHg is associated with mortality after ECPR (OR 1.77 (CI 1.03–3.03))
Chang et al. [17]	–	291	First P_a_O_2_ within 24 h	“Mostly from right radial artery” but data not available	12%	P_a_O_2_ between 77 and 220 mmHg is associated with favorable neurological outcome (OR 2.29 (CI 1.01–5.22))
Halter et al. [14]	–	66	P_a_O_2_ 30 min after ECPR start	Not available	62%	Hyperoxemia is associated with 28-day mortality (OR 1.89 (CI 1.74–2.07))
Ross et al. [13]	30	–	Mean P_a_O_2_ during the first 24 h	Right radial: 100%	43%	No association between P_a_O_2_ and mortality
Al Kawaz et al. [18]	90	42	Mean P_a_O_2_ during 24 first hours	Right radial: 100%	89%	Hyperoxemia is associated with in-hospital mortality (OR 1.18 (CI 1.08–1.29))
Bonnemain et al. [19]	–	44	Mean P_a_O_2_ during 24 first hours	Right radial: 47% Left radial: 18% Femoral: 30%	30%	Mean P_a_O_2_ is associated with mortality (OR 1.07 (CI 1.01–1.13))
Justus et al. [20]	41	11	Mean P_a_O_2_ during the entire ECMO support	Right radial: 100%	10%	No association between mean P_a_O_2_ and mortality
Stoll et al. [15]	–	79	≥ 1 episode of P_a_O_2_ > 300 mmHg during the first 8 days	Right radial: 100%	75%	Hyperoxemia is associated with 30-day mortality (OR 2.52 (CI 1.06–5.98))
Kashiura et al. [30]	–	847	First P_a_O_2_ after ECPR start	Not available	Not available	P_a_O_2_ > 400 mmHg is associated with 30-day neurological outcome (OR 0.48 (CI 0.29–0.82))
Kobayashi et al. [33]	–	110	P_a_O_2_ 24 h after ECPR start	Right radial or brachial: 100%	Not available	No association between mean P_a_O_2_ and 30-day mortality

CS, cardiogenic shock; ECPR, extracorporeal cardiopulmonary resuscitation; OR, odds ratio; CI, confidence interval

## Why targeting extracorporeal moderate hyperoxemia (P_POST_O_2_ 150 mmHg) during VA-ECMO?

The target of 150 mmHg for P_POST_O_2_ does not rely on randomized data. However, several observational and preclinical data support this recommendation [14, 17–19, 29, 30]. It might correspond to a safety zone, avoiding both hypoxemia and severe hyperoxemia.

### To avoid severe hyperoxemia

#### Hyperoxemia is associated with altered prognosis in VA-ECMO patients

Observational studies, including two pediatric ones, have reported an association between hyperoxemia (usually sampled on right radial artery) and outcomes in VA-ECMO patients [14, 15, 17–19, 29–33]. In these studies, severe hyperoxemia that is commonly defined by a P_a_O_2_ ≥ 300 mmHg is frequently associated with worst outcomes (Table 1). Despite well-known harmful effect of hyperoxemia, a causative link is still matter to discussion for several reasons. First, hyperoxemia definition was variable, with P_a_O_2_ threshold ranging from 101 to 301 mmHg. Second, identification of hyperoxemia was often based on only one arterial blood gas sample in four studies [14, 17, 29, 30]. As so, it represents a small window of oxygen exposure and does not analyze long-term exposure to hyperoxemia. In addition, the site of arterial blood gas sample differs between/within studies. Third, as VA-ECMO was mostly peripherally inserted, high P_a_O_2_ might reflect low native cardiac output and high level of circulatory support. In this setting, hyperoxemic patients might be those most severely ill [34]. Fourth, most of these studies analyzed ECPR patients who represent a specifically medical condition in which hyperoxemia seems particularly deleterious. Recently, a retrospective analysis of the ELSO database on 7488 ECPR patients showed that an increase in P_a_O_2_ between pre-ECMO and 24 h after ECMO start was associated with in-hospital mortality [7]. Of note, the respective role of hyperoxemia and hypocapnia secondary to the extracorporeal CO_2_ removal remains matter of debate [6, 7]. Indeed, the rapid decrease in P_a_CO_2_ induced by ECMO may also contribute to brain ischemia through cerebral vasoconstriction.

Despite a strong association between early hyperoxemia and death, there are few studies on the mechanism by which hyperoxemia may increase mortality in VA-ECMO patients.

### Hyperoxemia affects homeostasis and organ functions

Hyperoxemia induces radical oxygen species (ROS) production even in healthy volunteers exposed to inhaled oxygen [35]. During VA-ECMO, hyperoxemia might act as a booster of ROS production and reperfusion injury [36, 37]. In an experimental animal study, levels of TNF-α and IL-6 significantly increased with a P_a_O_2_ greater than 300 mmHg [38]. These findings suggest that hyperoxemia during VA-ECMO enhances systemic inflammation [39]. Severe hyperoxemia also reduced functional capillary density compared to extracorporeal normoxemia [40]. Taking together these phenomenon may contribute to organ dysfunction [41]. Because of shock and VA-ECMO support, ischemia–reperfusion injuries and hyperoxemia alter digestive mucosa barriers, which can be indirectly evaluated by the *Intestinal Fatty-Acid Biding Protein* (iFABP), a marker of enterocyte damage [42]. High iFABP values are associated with multi-organ failure and mortality [42–44]. In an experimental study on pigs supported by VA-ECMO, intestinal mucosa damage and intestinal permeability gradually increased with the duration of ECMO suggesting a role for the duration of hyperoxemia exposition [45, 46]. These results were confirmed by an animal study that demonstrated alteration of gut function in a dose- and time-dependent manner [44] with hyperoxemia. Although there are few clinical data on hyperoxemia during VA-ECMO and gut, it seems that hyperoxemia might enhance gut dysfunction secondary to VA-ECMO. These effects may explain the higher rate of bacterial translocation, and higher value of iFABP when rats are exposed to hyperoxemia [47].

Hyperoxemia has several positive and negative effects on cardiovascular system. Randomized studies in myocardial infarctions have reported conflicting results. While the AVOID trial demonstrated an increase in infarct size, arrhythmia occurrence, and recurrent infarction [48], the DETOX did not [49]. During cardiac surgery with cardiopulmonary bypass, hyperoxemia did not increase cardiovascular complications [50]. A retrospective study in cardiogenic shock after myocardial infarction supported by VA-ECMO did not demonstrate any harm of benefit of hyperoxemia [13].

VA-ECMO is often used for ECPR. In this context, hyperoxemia is potentially harmful. Observational studies have provided conflicting results on the effect of hyperoxemia on neurological outcomes. A randomized study has evaluated the neurological effect of mild hyperoxemia in 120 non-ECMO patients following cardiac arrest. Despite increasing tissue perfusion, hyperoxemia did not increase neuron-specific enolase value, a marker of neurological damage [51]. Equally, a post hoc analysis of the ICU-ROX trial did not demonstrate a decrease in poor neurological outcome at 6 months with conservative oxygen therapy [52].

### Hyperoxemia: a question of dose or time exposure, or both?

Despite several animals’ studies demonstrating harmful effect of hyperoxemia, randomized clinical studies during short-term exposure did not demonstrate these effects. Studies performed during cardiopulmonary bypass are of interest because they concern a specific population suffering of cardiovascular disease with controlled ischemia–reperfusion injury, and hyperoxemia. Thus, P_a_O_2_ up to 500 mmHg is not associated with worst cardiovascular, renal, and neurological outcomes [50, 53, 54]. For short-term exposure (*i.e., *during cardiopulmonary bypass), hyperoxemia may not be harmful [50, 54]. Another factor that we should consider may be the time exposure to hyperoxemia. Oxygen therapy is a drug for which studies demonstrated a dose effect and a time exposure effect. Several animals’ studies demonstrated this time exposure effect of hyperoxemia, particularly during ischemia–reperfusion process and systemic inflammation. Hyperoxemia may be a trigger that enhances the host response to injury. These findings were highlighted by a recent meta-analysis. By analyzing more than 5000 ICU patients, Ni et al. demonstrated that conservative oxygen therapy is associated with a shorter mechanical ventilation duration, a decrease in new organ failure during the ICU stays, and a lower risk of renal replacement therapy [55].

#### To avoid hypoxemia

The ELSO experts recommend avoiding extracorporeal hypoxemia, but they do not define a threshold value. In critically ill patients without ECMO, it is recommended to maintain S_a_O_2_ above 92% during mechanical ventilation [56]. Lower limits have even been tolerated in ARDS (S_a_O_2_ ≥ 88%, P_a_O_2_ ≥ 55 mmHg) [57, 58]. However, several recent randomized studies on oxygenation target have raised concern about possible harm with a P_a_O_2_ target lower than 70 mmHg compared to higher levels.

In a post hoc analysis of the ICU-ROX trial focusing on septic patients, there was a trend to higher mortality in the conservative oxygenation arm (pulse oximetry target: 90 to 96%) compared to usual care [4].

As well in the LOCO_2_ trial (ARDS patients), the mortality at 90 days was higher in the lower oxygenation arm (P_a_O_2_ 55 to 70 mmHg) [3]. Finally, a secondary analysis of the HOT ICU trial suggested a higher mortality in the lower oxygenation arm (P_a_O_2_ 60 mmHg) in the subgroup of patients with norepinephrine [5]. In summary, even if hyperoxemia should be avoided, P_POST_O_2_ should probably not be lower than 70 mmHg.

#### Because we cannot ensure strict extracorporeal normoxemia

Because of clot formation around the fibers of the membrane, oxygenation performance decreases over time [59]. In a retrospective study on 265 patients supported by veno-venous (VV)-ECMO, 10 patients had membrane lung exchange due to decreasing of gas transfer on oxygenator [60]. Consequently, F_S_O_2_ could not reliably predict P_POST_O_2_ over time, and for a constant FsO_2_, P_POST_O_2_ will decrease with time.

It is therefore theoretically necessary to measure continuously P_POST_O_2_ or S_POST_O_2_. As VA-ECMO blood flow is not pulsatile, pulse oximetry is unreliable to monitor S_POST_O_2_. Recently, three devices have been proposed to monitor membrane oxygenation: the LANDING ECMO™ (EUROSET), the System M4™ (SPECTRUM MEDICAL), and the NAUTILUS SMART™ (MEDTRONIC). While of potential interest, these devices are currently not widely available. Furthermore, their reliability has to be tested during prolonged usage. Waiting for such a continuous monitoring system, direct measurement of P_POST_O_2_ is probably useful at least once a day to rule out severe hyperoxemia and hypoxemia. Attention should also be paid to variation of oxygen transfer determinants (*i.e.,* F_S_O_2_, hemoglobin concentration, and ECMO blood flow), which could result in significant change of P_POST_O_2_, and need to repeat the measure.

Finally, it should be kept in mind that a severe P_POST_O_2_ drop (resulting in postoxygenator hypoxemia) will be detected by continuous monitoring of near-infrared spectroscopy (NIRS) of the cannulated limb. Indeed, as the cannulated limb oxygenation is totally determined by the oxygenator, a sudden drop of NIRS value indicates reperfusion cannula occlusion, insufficient blood flow, or postoxygenator hypoxemia.

### Landscape of the needed and current studies about extracorporeal oxygenation during VA-ECMO support

Needed and current studies about extracorporeal oxygenation during VA-ECMO support are summarized in Table 2.

**Table 2:** Needed and current studies about extracorporeal oxygenation during VA-ECMO support

Accuracy of a continuous monitoring of S_POST_O_2_
International observational study of extracorporeal oxygenation practice during VA-ECMO
Identification of the oxygenation determinants during VA-ECMO support
Feasibility of a normoxemic extracorporeal strategy in VA-ECMO (NCT04990349, ECMOXY)
Efficacy of a normoxemic extracorporeal strategy in VA-ECMO (NCT03841084, BLENDER)
Efficacy of a normoxemic extracorporeal strategy in VA-ECMO (French PHRC 2022, ECMOX2)

## Conclusion

Defining extracorporeal oxygenation targets for VA-ECMO patients remains challenging, as there is no published randomized trial. Data from observational studies are limited by their design and the definition of hyperoxemia. There is a need to define oxygenation targets for the right radial P_a_O_2_ and the P_POST_O_2_. Pending specific data on ideal oxygenation targets during VA-ECMO support, avoiding both hypoxemia and severe hyperoxemia, seem reasonable.

## Data Availability

Not applicable.

## Abbreviations

ELSO: Extracorporeal Life Support Organization
VA-ECMO: Veno-arterial extracorporeal membrane oxygenation
ECPR: Extracorporeal cardiopulmonary resuscitation
S_a_O_2_: Arterial oxygen saturation of hemoglobin
P_a_O_2_: Arterial oxygen partial pressure
F_I_O_2_: Inspired oxygen fraction
S_v_O_2_: Mixed venous oxygen saturation of hemoglobin in the pulmonary artery
SVC: Superior vena cava
S_SVC_O_2_: Oxygen saturation of hemoglobin in the superior vena cava
IVC: Inferior vena cava
S_IVC_O_2_: Oxygen saturation of hemoglobin in the inferior vena cava
P_POST_O_2_: Postoxygenator oxygen partial pressure
S_POST_O_2_: Postoxygenator oxygen saturation of hemoglobin
S_PRE_O_2_: Preoxygenator oxygen saturation of hemoglobin
F_s_O_2_: Sweep gas oxygen fraction
ROS: Radical oxygen species
iFABP: Intestinal fatty acid-binding protein
